# Metabolomics in Alcohol Research and Drug Development

**Published:** 2008

**Authors:** George G. Harrigan, Greg Maguire, Laszlo Boros

**Keywords:** Alcohol abuse and dependence, alcoholism, alcohol-related disorders, systems biology, metabolomics, metabolome, metabolic networks, metabolic pathways, metabolites, metabolic process, metabolic profiling, lipid dysfunction, thiamine deficiency, disease etiology, drug design

## Abstract

Developers of new medications need to describe and predict the functional attributes of test compounds administered to cells, animals, and humans. Today, researchers increasingly appreciate the role that intermediary products (i.e., metabolites) generated in the course of various metabolic pathways play in both health and disease states and how their analysis can support development of new medications. Advances in analytical and computational techniques have facilitated the rise of new and powerful tools for measuring metabolic and biochemical pathways in such complex systems. Metabolomics—a systems biology approach to characterizing metabolites produced in biochemical pathways—is contributing to many studies of disease progression and treatment, although it has not yet been extensively applied in research on metabolic perturbations associated with alcohol abuse. However, numerous metabolomic approaches may contribute to alcohol-related research, as illustrated by studies on alcohol-related metabolic dysfunctions such as (1) alterations in fat metabolism and (2) thiamine deficiency. By further increasing the number and types of metabolites that can be measured in a given biological sample, metabolomic approaches may be able to help define the role of the many different metabolic pathways affected by alcohol abuse and support discovery and development of novel medications for the treatment of alcoholism and related conditions.

Attempts to develop new and/or improved medications for the treatment of illnesses affecting millions of people around the world are characterized by an ever-increasing disparity between research expenditure (both in terms of time and money) in the pharmaceutical industry and return on investment (Di Masi et al. 2003). Therefore, strategies that can rapidly identify novel candidate medications (or potential targets of new medications), which are likely to demonstrate effectiveness in clinical trials, will be valuable to both patients and the pharmaceutical industry. One means to achieve this goal—the adoption of screening approaches that can be performed in isolated cells and tissues (i.e., in vitro) or using computer models (i.e., in silico)—continues apace. Another important prerequisite for effective screening tools is the development of analytical and computational methodologies to describe the effects of medication candidates within the complex biochemical pathways of intact, fully assembled living networks (Goodacre et al. 2004; Joyce and Palsson 2007; Maguire et al. 2006, 2007; Mo et al. 2007). A most promising approach to studying such networks is metabolomics.

Metabolomics is a systems biology approach to measuring changes in small-molecule intermediary products (i.e., metabolites)—such as sugars (e.g., glucose), amino acids, and organic acids—in any given biological system exposed to genetic modification, drugs or medications, or any other environmental perturbation. The levels of these small-molecule metabolites within a cell, tissue, or an organism largely reflect the endogenous activity of biochemical pathways; moreover, they also are highly responsive to external or environmental changes. Identification of induced changes in the concentration of these metabolites, or altered flux^1^ rates through metabolic pathways (e.g., Maguire et al. 2006; 2007), may provide insights into the activities, interactions, and regulatory roles of all biochemical processes of interest.

Although metabolomic approaches to date have been used primarily for other physiological processes and diseases, they are equally applicable to studies on the impact of alcohol on metabolism as well as the effect of medications developed to treat the effects of alcohol abuse and dependence. The costs associated with alcoholism, at both the personal and more global level, are well understood. From a medical standpoint, it is important both to understand the psychological and emotional factors that contribute to alcohol dependence and to ameliorate the physiological devastation that this dependence can cause. Because excessive alcohol use can induce profound metabolic perturbations, there should be numerous opportunities for using metabolomic approaches. Indeed, the first attempts at such metabolic profiling research date back to at least the 1980s, when researchers measured the levels of various metabolites in the urine of ethanol-exposed humans and rats (Axelson et al. 1981; Weiner et al. 1984). More recently, however, metabolomic strategies rarely have been applied systematically in the alcohol field.

This article briefly describes the concepts behind metabolomic research. It highlights examples of how metabolomic approaches have been used to study metabolic perturbations, such as altered fat (i.e., lipid) metabolism and thiamine deficiency, that also are observed in alcohol-dependent patients. It also considers the role that metabolomics can play in further elucidating the causes and effects of these changes and supporting the discovery and development of new medications to treat alcoholism and its metabolic consequences.

## What Is Metabolomics?

Many definitions of metabolomics exist (Fiehn 2002; Goodacre et al. 2004), but, in general, the principal goals of metabolomics are to probe the mechanisms that contribute to changes in metabolite levels and fluxes in cells, tissues, and organisms and to understand the implications of such changes on disease, response to drugs and medications, and nutritional intervention. To achieve these goals, numerous technical approaches to data acquisition are available. The main technologies utilized in metabolomics are nuclear magnetic resonance (NMR) spectroscopy and mass spectrometry (MS) (for a review, see Dunn and Ellis 2005). Briefly, NMR spectroscopy is a technique that exploits the magnetic properties of the nuclei of certain atoms (e.g., protons [^1^H] or a variant [i.e., isotope] of carbon known as ^13^C). In a typical metabolomics experiment, a solvent extract of the biological sample (or in the case of urine or plasma, the sample itself) is placed in a magnetic field and then exposed to radiowaves covering a given frequency range. The way in which the biological sample absorbs that energy is dependent on the relative proportion of different metabolites in that sample. This allows investigators to obtain an NMR spectrum that represents the metabolite composition of the sample and that can be compared with the NMR spectra of other biological samples.

MS is another analytical technique that can be used to assess the metabolite composition of a biological sample. For these assays, samples are injected into a mass spectrometer that can separate compounds according to their molecular mass, and a list of the molecular masses of the compounds in the sample is generated. The metabolites corresponding to a given molecular mass can then be identified and their relative abundance in a sample determined.

The specific data acquisition technology used typically depends on the nature and biochemical composition of the metabolites to be measured. Thus, MS may represent a better option to measure certain classes of metabolites, whereas NMR may be the better choice for others. In general, MS methods are more sensitive than NMR and can identify molecules that are present in only small amounts.

NMR and MS, as well as other techniques, can be employed in two different (but not necessarily mutually exclusive) conceptual approaches that can broadly be defined as “non-targeted” and “targeted” (Goodacre et al. 2004). Nontargeted approaches provide a hypothesis-free global overview of readily detectable metabolites in a sample (e.g., lipid or sugar metabolites in plasma). Many experiments have successfully used such nontargeted approaches, although some investigators are increasingly concerned about current statistical treatments and methodological practices in such experiments (Broadhurst and Kell 2006; Lay et al. 2006). It has been suggested, for example, that the results of a study claiming that nontargeted NMR analysis of plasma could accurately predict the risk of coronary heart disease actually were confounded by gender and drug use (Kirschenlohr et al. 2006).^2^

Targeted approaches, in contrast, focus on identified and preselected metabolic pathways, which may offer particular advantages in studies on metabolic dysregulation. These targeted approaches typically employ optimized measurement techniques that specifically detect certain classes of metabolites or pathways. This approach recognizes the wide differences in physiochemical structure, stability, and abundance of different metabolites in a given sample—factors that present challenges in accurately measuring a wide range of metabolites in a single measurement. Targeted approaches also recognize that evaluation of low-abundance metabolites, such as signaling lipids, hormones, and neurotransmitters, may require specialized methods. Finally, targeted metabolomic approaches emphasizing biological expertise in experimental strategy can be used in flux-based methodology to determine the fate of certain metabolites. For example, specifically designed substrates that have been labeled with easily measurable tracers (e.g., radioactive molecules) can be introduced into test biological systems and their distribution and metabolic fate recorded (Hellerstein and Murphy 2004). To date, flux-based approaches unfortunately are underrepresented in metabolomics.

### Advantages of Metabolomics

Metabolomic approaches have several advantages that can help them complement other strategies, such as genomic, transcriptomic, and proteomic approaches. First, many examples have demonstrated that the presence of a gene which has been shown to be associated with a disease need not always lead to disease manifestation, and in many cases further biological information is required to predict the onset of symptoms. The presence of a certain variant (i.e., mutation) of the gene associated with Huntington disease, for example, clearly correlates with neural degeneration but does not necessarily predict when the first preclinical symptoms will appear. To identify biomarkers that can evaluate or predict the onset of certain diseases, it therefore may be more promising to use a combinatory approach that associates the presence of a specific, predisposing genetic variant with the presence of altered metabolite patterns in the blood. Similarly, metabolomic approaches may be useful in determining the risk of diseases that are determined by several genes (i.e., are polygenic in origin), such as alcohol dependence. In these cases, the contribution of a single gene to disease variance likely is small and/or difficult to assess, and integrating information on a single gene variant with transcriptomic, proteomic, and metabolomic data may prove informative.

Second, metabolomic approaches can be useful because disease variation depends not only on the effects of genetic factors but also is influenced by environmental factors. For example, a person’s diet profoundly impacts his or her lifespan and resistance to disease. Moreover, certain diseases, including alcohol dependence, frequently go hand in hand with inadequate nutrition. It therefore is reasonable to envision a role for metabolomics in dissecting the relative contributions of these nutritional factors to disease susceptibility or progression. Indeed, metabolomics now is considered an important discipline among researchers concerned with the effects of nutrition, as exemplified by initiatives by the European Nutrigenomics Organization (http://www.nugo.org). Similarly, the NIH Roadmap Initiative (http://nihroadmap.nih.gov/initiatives.asp) and the NIH Genes, Environment and Health Initiative (http://www.gei.nih.gov) include metabolomic initiatives that, among other goals, aim to determine the influence of environmental factors on disease susceptibility and progression.

Third, as is the case with the presence of specific genes, the presence of certain messenger RNAs (mRNAs) or proteins does not always suffice to predict disease onset. In fact, the correlation between global mRNA levels and protein expression typically is low (i.e., production of large amounts of mRNA does not automatically imply production of large amounts of protein). Furthermore, despite some successes, it appears that many biomarkers initially identified through transcriptomics or proteomics cannot be validated (Lay et al. 2006). Although part of this failure may be related to technical issues, it also is known that changes in, for example, the levels of individual enzymes need not correlate with changes in metabolic flux or overall function of the cell or tissue (Kacser 1995). Thus, transcriptomics and proteomics analyses may not suffice to fully assess genetic or environmental contributions to disease variance. Indeed, the metabolome may represent the functional status of a cell, tissue, or organism more closely because it reflects the overall effects of transcriptomic and proteomic changes. Moreover, because the metabolome is the “downstream” result of gene expression, changes in the metabolome are amplified relative to changes in the transcriptome and the proteome, thus making measurements more tractable.

In summary, because the metabolome is reasoned to be more directly related to cell or organ status than the transcriptome or proteome, and is responsive to a wide range of perturbations, it represents a viable option for increasing our understanding of the factors that influence development and progression of various diseases. Accordingly, metabolomic analyses may help elucidate the dynamics of alcohol dependence and of alcohol-induced organ damage as well as clarify the role of factors such as genetic predisposition and nutritional deficiencies or the effects of new medications.

## Relevance of Metabolomics to Alcohol Research

Biochemical studies have contributed greatly to our understanding of metabolic perturbations in alcohol-dependent patients. For example, alcohol’s impact on lipid metabolism in different organs increasingly is understood (Baraona and Lieber 1998; Crabb and Liangpunsakul 2006). Similarly, alcohol-related dysfunction in carbohydrate metabolism has been well-studied, and particularly the importance of the vitamin thiamine (which serves as a co-factor for at least three enzymes involved in carbohydrate metabolism) has become evident in studies focusing on alcohol-induced damage to the nervous system (i.e., neurodegeneration) (Martin et al. 2003). Studies of other disorders have already demonstrated that the concentrations, fluxes, and transport mechanisms of small-molecule metabolites generally are highly sensitive to disease. Because alcohol-induced metabolic perturbations obviously are a major contributing factor to disease progression, metabolomics may play a role in dissecting the contributions of different biochemical pathways to the development of alcohol-related damage.

To date, there have been few examples of metabolomic research specifically applied to alcohol-related disorders. However, there are certainly potential opportunities for metabolomic research in the alcohol field, particularly targeted and flux-based approaches, as illustrated by two examples of studies of known metabolic effects associated with alcohol-related diseases. The following sections review two major areas of metabolic dysregulation that are caused by numerous diseases or nutritional deficiencies, including alcohol abuse and dependence, and for which application of metabolomic approaches has provided new insights. These are (1) lipid dysfunction, which is associated with liver (i.e., hepatic) and cardiovascular disease; and (2) thiamine deficiency and dysfunction in carbohydrate metabolism, which is associated with, among other things, neurodegeneration.

### Alcohol-Associated Lipid Dysfunction

Studies have suggested that alcohol (chemically known as ethanol) may interfere with the processes that regulate the balance in the body between lipid breakdown as a source of energy and lipid synthesis. Lipid metabolism can be profoundly altered in alcohol-dependent patients (Crabb 2004; Crabb and Lianpunsakul 2006). As with many disease states, nutritional influences, and medications, alcohol interferes directly with the use of fatty acids as a source of energy in the liver. (For information on the general structure of lipids, see sidebar, below.) As a result, triglycerides accumulate in the liver. These triglycerides also are secreted into the blood, contributing to elevated levels of circulating lipids (i.e., dyslipidemia).

These processes of lipid synthesis and breakdown are regulated by several factors that control the activation (i.e., transcription) of genes involved in lipid metabolism. Tw o of these factors are the peroxisomal proliferator–activated receptors (PPARs) and the sterol response element–binding proteins (SREBPs)-1 and -2. PPARs are hormone receptors located in the cell nucleus, and if they do not function properly, disorders in lipid metabolism, as well as insulin resistance, may develop. SREBPs act as “sensors” for fatty acid and cholesterol levels in blood and tissue, thereby helping to maintain constant levels (i.e., homeostasis) of lipids in the body. Broadly speaking, activation of PPARs enhances conversion of lipids into energy, whereas activation of SREBP-1 induces an opposing effect. Studies conducted in ethanol-exposed cultured cells of mice suggest that ethanol interferes with transcription-activating properties of one type of PPAR (i.e., PPARα) but activates SREBP-1 (Crabb 2004). Both of these effects would result in lipid accumulation in the blood and tissues and could contribute to alcohol-related liver damage (i.e., fatty liver).^3^

**Structure and Metabolism of Lipids**

Lipids play numerous important functions in the body. In addition to serving as energy stores, they are essential components of all cell membranes. Chemically, lipids are water-repellent (i.e., hydrophobic) molecules that include fats, oils, waxes, sterols (e.g., cholesterol), and other related compounds.

From a metabolic and physiological standpoint the triglycerides represent one of the most important lipid classes. Each triglyceride molecule is composed of the following molecules.

- Glycerol—an alcohol containing three carbons, each of which is linked to a hydroxyl group (OH); and
- Three fatty acids—chains of varying length made up of carbon and hydrogen atoms with a carboxyl group (COOH) at one end that can link the fatty acids to the glycerol molecule.

Fatty acids fall into two main groups: saturated or unsaturated. Saturated fatty acids (which are found primarily in animal sources) contain the maximum number of hydrogen atoms (i.e., two hydrogens per carbon). Unsaturated fatty acids (which are found primarily in plant sources), in contrast, lack one or more hydrogen atoms and therefore have one or more “double-bonds” between two carbons (see figure).

For both saturated and unsaturated fatty acids the length of the hydrocarbon chain (i.e., the number of carbon atoms) can differ, which can affect their metabolism as well as their specific function in the body.

For triglycerides to be metabolized as energy sources, the fatty acids are first separated from the glycerol. The free fatty acids then are transported to the mitochondria of the appropriate cells, where they are sequentially broken down to generate an intermediary molecule called acetyl-CoA, which can enter the citric acid cycle that also is involved in glucose metabolism. (Conversely, acetyl-CoA can be diverted from glucose metabolism for fatty acid and lipid synthesis.) In addition, fatty acids can be involved in numerous other metabolic reactions (e.g., be converted from unsaturated to saturated fatty acids or incorporated into longer chain fatty acids).

**Figure:**
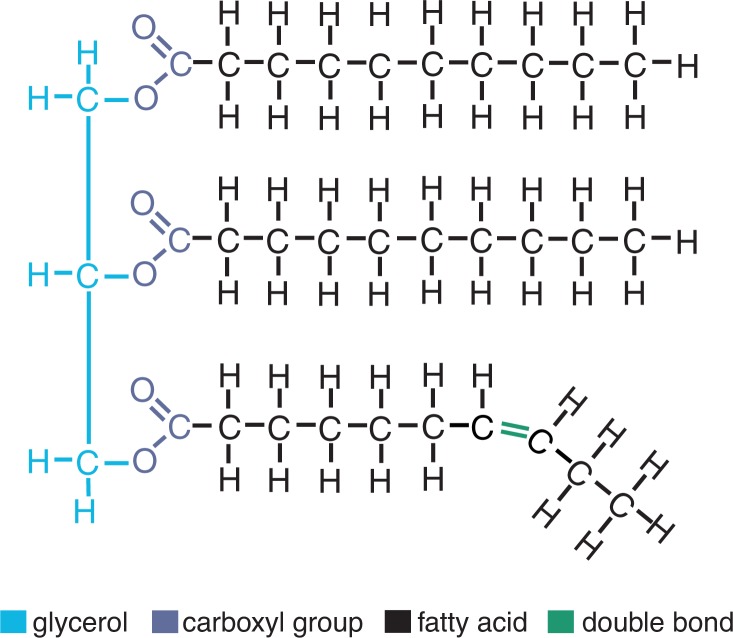

Fat and liver cells can synthesize and store triglycerides. Alcohol can increase levels of triglycerides in the liver. Increased levels of triglycerides in the blood are associated with atherosclerosis and increased risk of heart disease.

—George G. Harrison, Greg Maguire, and Laszlo Boros

Altered lipid metabolism resulting from alcohol exposure also can lead to the generation of highly reactive, oxygen-containing small molecules known as reactive oxygen species (ROS), which can cause damage in a variety of tissues and further exacerbate disease progression (Lieber 2004). Accordingly, a further understanding of how the levels and metabolism of lipids are regulated may contribute to the development of therapies for alcohol-related disorders. In fact, several studies already have indicated that medications which activate PPAR (and thereby counteract the effects of ethanol), such as the medication pioglitazone, can ameliorate fatty liver in ethanol-fed rats.

Methods for measuring lipids continue to develop and improve. Many now allow both the quantitative analysis of lipid classes present in a sample and determination of the entire fatty acid composition within those classes (Mutch 2006). This latter aspect is important because by determining which specific fatty acids serve as substrates for distinct metabolic pathways, researchers can obtain further information on specific enzymes and other regulatory mechanisms that control lipid synthesis, transport, and distribution. This knowledge then can improve the understanding of both disease progression and response to therapeutic intervention. For example, it has been suggested that liver lipids of alcohol-dependent patients contain a higher ratio of unsaturated fatty acids than those of nonalcoholic control subjects (Bunout 1999). This observation implies that alcohol-dependent patients have an elevated activity of a certain enzyme called Δ9-desaturase that has been associated with metabolic disorders.

Compositional analyses of the lipids in a tissue or organ can be complemented by flux analyses that assess the distribution and metabolism of lipids in the body. For example, Pawlosky and Salem (1999) evaluated the effect of ethanol on the metabolism of two polyunsaturated fatty acids—linoleic acid and α-linolenic acid—in Rhesus monkeys. In this study, alcohol consumption did not appear to affect the absorption of these fatty acids into circulation. However, the incorporation of these fatty acids into longer-chain molecules was increased, suggesting that alcohol enhances the elongation of polyunsaturated fatty acids.

Other investigators found that alcohol also interferes with the synthesis and transport of long-chain fatty acids. Boros and colleagues (2002) studied the de novo synthesis of the fatty acid palmitate, its elongation to stearate, and the conversion (i.e., desaturation) of stearate into oleate in the liver and pancreas of rats who were fed alcohol directly into the stomach for 4 weeks. To determine the levels of these fatty acids, the researchers provided the animals with water containing the stable hydrogen isotope deuterium, which was then incorporated into the newly synthesized fatty acids and their metabolites and could be measured using MS. These analyses found that compared with control animals, synthesis of palmitate, stearate, and oleate in the liver (expressed as the percentage of total fatty acid synthesis in the liver) decreased significantly in the alcohol-treated animals. In the pancreas, in contrast, palmitate and stearate synthesis also decreased to a similar extent, whereas oleate synthesis actually increased compared with control rats. Additional analyses indicated that in the liver of alcohol-treated animals, stearate accumulated relative to palmitate (i.e., elongation of palmitate proceeded effectively) but that oleate levels relative to palmitate decreased (i.e., desaturation was impaired). In the pancreas, however, the opposite effect of alcohol exposure was noted—the ratio of stearate to palmitate decreased, whereas the oleate-to-palmitate ratio increased in the alcohol-treated animals compared with the control animals. Together, these findings indicate that long-term alcohol exposure increases the fatty acid content of liver cells, decreases de novo synthesis of fatty acids, and has opposite effects on fatty acid accumulation in the liver and pancreas. Because adequate levels of unsaturated fatty acids such as oleate are critically important for various physiological processes (e.g., the transport of long-chain saturated fatty acids out of the liver), the decreased oleate ratio in the liver of alcohol-treated animals can result in decreased fatty acid transport and may contribute to fatty liver. In addition, the increased oleate ratio in the pancreas promotes processes that may support the rapid development of pancreatitis.

Metabolomic approaches such as lipid profiling also may contribute to the development of safe medications to treat alcohol-induced dyslipidemia. For example, in a recent study on the PPAR activator, rosiglitazone, in a diabetic mouse model the researchers found reduced levels of cholesterol and triglyceride levels in the blood, suggesting that the medication led to a decrease in new lipid synthesis. Additional analyses, however, demonstrated that rosiglitazone treatment actually resulted in fat accumulation in the liver and tissue toxicity (Watkins et al. 2002). This may imply that new medications designed to modulate lipid metabolism should be evaluated by comprehensive metabolomics investigations extending beyond measurements of only one or two targeted lipid metabolites.

Lipids clearly are an extremely important component of the metabolome, and further developments in data acquisition technologies can only be of marked benefit. Recognizing this, the U.S. National Institute of General Medical Sciences has, since 2003, sponsored a LIPIDMAPS consortium of 16 academic research institutes and two private enterprises to fully map metabolic pathways in a type of immune cell, the macrophage. The goals of LIPIDMAPS (www.lipidmaps.org) are to (1) separate and detect all lipids in a specific cell and discover and characterize any novel lipids that may be present, (2) quantitate each of the lipid metabolites present and quantitate the changes in their levels and location during cellular function, and (3) define the biochemical pathways for each lipid and develop lipid maps that define the interaction networks. Because macrophages play, among other functions, a significant role in the formation of atherosclerotic lesions, understanding and defining changes in macrophage biology in terms of changes in lipid metabolism will prove invaluable to the discovery of new medications.

### Thiamine Deficiency and Flux Analysis of Transketolase Activity

Thiamine, also known as vitamin B1, is essential for the optimal functioning and health of the cardiovascular, nervous, and digestive systems. Alcohol-dependent patients frequently exhibit abnormally low levels of thiamine in the blood that result mainly from nutritional deficiencies, from alcohol’s impact on thiamine absorption from the gastrointestinal tract, and from impaired thiamine utilization in cells (Bridges et al. 1999). The abnormally low levels of thiamine are thought to contribute to the neurodegeneration often seen in alcohol-dependent patients (Martin et al. 2003). Indeed, some evidence suggests that the degeneration of a brain region called the cerebellum, which often is associated with alcohol dependence, is associated more with the toxic effects of thiamine deficiency than with the direct toxic effects of alcohol itself (Mulholland et al. 2005).

Thiamine plays a key role in carbohydrate metabolism and is indeed essential for the conversion of carbohydrates into energy because it is a co-factor required by three enzymes—transketolase, pyruvate dehydrogenase (PDH), and α-ketoglutarate dehydrogenase (KGDH)—that mediate various steps in the breakdown of glucose (see sidebar, p. 32).

Transketolase plays a role in a chain of reactions called the pentose phosphate pathway, whereas PDH and KGDH are involved in glycolysis and the citric acid cycle. Accordingly, it appears plausible that metabolomic strategies that can help analyze carbohydrate metabolism also may contribute to a further understanding of the role of alcohol-dependent thiamine deficiency. Moreover, such strategies could also, at least in principle, assist in developing approaches to identify people who are at particularly high risk of alcohol-related neurodegeneration.

Flux-based analyses that can monitor the distribution of ingested metabolites (e.g., glucose) and their metabolites in the cells and tissues are particularly useful in studies on carbohydrate metabolism. To this end, biological samples to be studied are treated with substrates that can be traced easily—for example, because they are labeled with a tracer (e.g., the stable, non-radioactive isotope ^13^C) that serves to differentiate the substrate from endogenous metabolites. As the tracer-labeled substrate is metabolized in the biological sample, the label will be transferred to other molecules that then can be further followed and identified. In the case of ^13^C-labeled substrates, MS can be used to reveal the positions that the tracer isotope assumes in metabolite products derived from the original tracer-labeled substrate. These analyses can reveal the specific reactions and pathways in which the original tracer-labeled substrate was involved. This metabolomic approach of monitoring metabolic pathways and reactions step-by-step (also known as stable isotope dynamic metabolic profiling [SIDMAP]) allows researchers to conduct comprehensive and disease-specific metabolic profiling in a dynamic, living, functioning cell or organism.

[1,2-^13^C_2_]-D-glucose is a particularly useful metabolite for studies on carbohydrate metabolism. It is especially helpful for discriminating between glucose metabolism through the oxidative and nonoxidative branches of the pentose phosphate pathway (see sidebar, p. 32). It was used, for example, in a study of a blood disorder called thiamine-responsive megaloblastic anemia (TRMA), which is characterized by dysfunctional red blood cells in the bone marrow. The study demonstrated that the fundamental defect in TRMA is impaired transketolase activity, which subsequently leads to decreased nucleic acid synthesis and disruption of the cell’s normal progression through the cell cycle (Boros et al. 2003). Commenting on this work in the same journal issue, Green (2003, p. 3,464) remarked that metabolomics has “made it possible to unlock doors along previously inaccessible hallways of gene function analysis” and that metabolic flux analysis provided a “key to better understanding of changes in substrate flow as a basis for drug mechanisms and disease.”

**Different Pathways of Glucose Metabolism**

The metabolism of the sugar glucose is one of the central metabolic processes in the body. It serves to provide energy to the body, as well as to provide starting materials for other metabolic pathways (e.g., the production of nucleic acids, fatty acids, and neurotransmitters). In addition, other metabolic pathways also feed into the reactions that occur during glucose metabolism, further emphasizing the central roles that the reactions involved in glucose metabolism play in ensuring the body’s functioning and well-being.

Glucose metabolism can proceed along several alternative pathways, depending on the body’s needs at the time. The following are three main sets of reactions associated with glucose metabolism (see figure):

- Glycolysis;
- The citric acid cycle; and
- The pentose phosphate pathway

During glycolysis, glucose is converted in several reactions into a molecule called pyruvate, which can be either metabolized further into lactate, that is then transported out of the cell, or which stays in the cell. Pyruvate is converted (through the actions of the thiamine-dependent enzyme pyruvate dehydrogenase) into a molecule called acetyl-CoA, which can then be fed into the second set of reactions, the citric acid cycle (also known as the tricarboxylic acid [TCA] cycle). Alternatively, acetyl-CoA may serve as a precursor for fatty acid synthesis (see sidebar, p. 29).

In the subsequent citric acid cycle, the acetyl-CoA is linked to a compound called oxaloacetate to form citric acid. The latter is then metabolized in a series of steps to form α-ketoglutarate, which is then transformed further to yield oxaloacetate again so that the cycle can continue. These reactions also involve a thiamine-dependent enzyme, α-ketoglutarate dehydrogenase. In addition, the various reactions of the citric acid cycle yield several metabolites that either serve as cofactors in other biochemical reactions or can be fed into other reactions yielding energy required by the cell to function normally.

The citric acid cycle plays a central role not only in carbohydrate (i.e., glucose) metabolism but also in protein and lipid metabolism, because both amino acids and fatty acids can be broken down into acetyl-CoA that enters the citric acid cycle.

The pentose phosphate pathway of glucose metabolism is an alternative pathway to glycolysis and the citric acid cycle that serves to generate both a co-factor that is required by many enzymatic reactions (reduced nicotinamide adenine dinucleotide phosphate [NADPH]) and sugar molecules containing five carbons (rather than the six-carbon sugars, such as glucose), which are needed, for example, for nucleic acid synthesis. The pentose phosphate pathway has two distinct phases, an oxidative phase, during which the NADPH is generated (as well as sugar called ribulose-5-phosphate), and a nonoxidative phase, during which the ribulose-5-phosphate is converted further into other five-carbon sugars. The nonoxidative phase also can serve as a “link” to glycolysis by generating fructose-6-phosphate and glyceraldehyde-3-phosphate. Several reactions of the nonoxidative phase involve the thiamine-dependent enzyme transketolase.

The relative contributions of all these pathways to glucose metabolism depend on the body’s needs at the time.

—*George G. Harrison, Greg Maguire, and Laszlo Boros*

**Figure:**
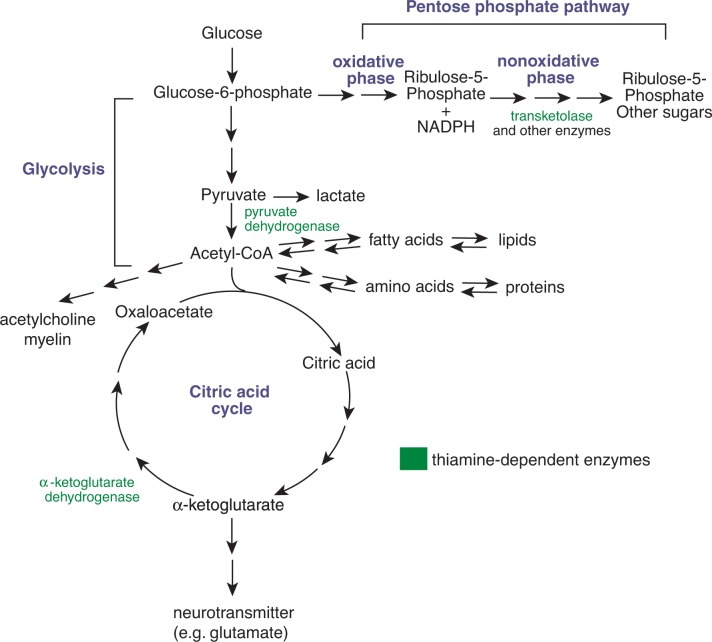

The value of this technology for understanding disease progression and aiding medication development also can be illustrated using the example of a recent investigation of the mechanism of action of the medication imatinib mesylate (Gleevec^®^), a cancer drug used in the treatment of a certain type of leukemia. Although Gleevec itself has no proven application in alcohol-based research, its SIDMAP analyses illustrate how metabolic flux in several pathways known to be affected by alcohol abuse can be measured simultaneously.

The leukemia cells against which Gleevec is active are characterized by increased glucose uptake, enhanced activity of an enzyme called hexokinase, and increased activity of the oxidative branch of the pentose phosphate pathway, all of which result in synthesis of new nucleic acids that are necessary for the leukemia cells to multiply. Gleevec acts by inhibiting all of these processes, thereby preventing further nucleic acid synthesis. However, in some patients the tumor cells become resistant to Gleevec treatment. SIDMAP approaches now have shown that cells develop Gleevec resistance by enhancing the activity of the non-oxidative portion of the pentose phosphate pathway (which involves the transketolase enzyme), which is not appreciably inhibited by Gleevec.

One of the greatest advantages of this metabolomic approach, however, is that it can be performed not only in cultured cells but also in the intact organism (i.e., in vivo). This means that this type of analyses can, for example, be performed in peripheral blood cells during Gleevec treatment of human patients. In this situation, SIDMAP analyses of oxidative versus nonoxidative pentose metabolism potentially may help clinicians predict the effect of Gleevec therapy and provide early indications of resistance to this treatment. Thus, the SIDMAP technology can contribute to basic research and early medication development as well as to clinical applications in which the activity and interactions of a multitude of defined biochemical pathways can be quantified (Maguire et al. 2006).

It is likely that metabolomic approaches such as SIDMAP also can be applied in alcohol research—for example, for assessing the effects of thiamine and thiamine deficiency not only on a single metabolic pathway but also on an integrated metabolic network. For example, thiamine-induced activation of transketolase activity helps control the levels of a glucose-derived molecule (i.e., sorbitol) that, at elevated levels, can cause complications in patients with diabetes (Berrone et al. 2006). Accordingly, reduced transketolase activity resulting from alcohol-induced thiamine deficiency may contribute to complications in alcoholic diabetic patients. Strategies, such as the one described above, that can analyze multiple metabolic pathways may therefore provide a more complete picture of the metabolic dysregulation associated with alcoholism and other conditions and may help predict better the effect of new medications in the treatment of this dysregulation.

## Other Recent Developments

The above examples have illustrated the potential benefits of metabolomics for analyzing two metabolic pathways—lipid metabolism and thiamine-dependent carbohydrate metabolism—that are affected by, or contribute to, the harmful effects associated with alcohol dependence. However, other metabolic pathways and metabolite classes, as well as the technologies for their unequivocal identification and quantitation, also may be relevant to alcohol research.

For example, another interesting potential application of metabolomic studies of carbohydrate metabolism to alcohol research is based on observations suggesting that breakdown products of glucose that are generated during the citric acid cycle (see sidebar figure, p. 92) and which circulate in the blood may play a role in hypertension. It is well-established that the prevalence of hypertension is higher among alcohol-dependent patients than among the normal population (Kodavali and Townsend 2006). The citric acid cycle takes place in special membrane-enclosed compartments (i.e., organelles) in the cells called mitochondria. These organelles often are referred to as the cell’s energy factories because the citric acid cycle and other late steps of glucose metabolism and the resulting energy production occur there. Two intermediary molecules generated during the citric acid cycle are succinate and α-ketoglutarate. It has been reasoned that, at least in animal models, these two compounds can increase blood pressure when secreted into the blood (He et al. 2004). Other mitochondrial metabolites also have been found to play a signaling role for other physiologic functions (MacDonald et al. 2005), and this research area clearly offers opportunities for targeted metabolomics of mitochondrial products.

Applying such an approach, Sabatine and colleagues (2005) demonstrated that the levels of functionally related metabolites of the citric acid cycle circulating in the blood differed between healthy people and people who were susceptible to constriction of blood vessels and reduced blood flow (i.e., ischemia) when exercising. The investigators demonstrated that the circulating levels of two citric acid cycle intermediates (i.e., oxaloacetate and citrate) decreased in ischemia-susceptible but not control subjects. The same was true for other compounds that are either end products of reactions related to the citric acid cycle or which enter the citric acid cycle through other metabolic pathways. In contrast, the levels of lactic acid (which is produced as an alternative product during glucose metabolism if the citric acid cycle is not used) greatly increased in both ischemia-susceptible and healthy subjects. As this example demonstrates, analyses using such comprehensive platforms that allow investigators to unequivocally identify metabolites or to survey metabolites potentially can provide insight into functionally relevant mechanisms contributing to disease progression (in this case, hypertension or ischemia).

Additional studies will have to determine whether these differences in mitochondrial metabolic products between people susceptible to ischemia and other people also may be in some way related to the elevated risk of hypertension in alcoholics. This notion is supported by the observation that excessive alcohol use may lead to damage of the mitochondria and may promote oxidative stress, particularly in liver cells, thereby contributing to liver damage (Hoek et al. 2002). Accordingly, it is conceivable that alcohol’s adverse effects on the mitochondria also may be associated with other harmful consequences, such as hypertension.

Finally, metabolomic approaches also may be of use for discovering biomarkers of alcohol abuse. One biomarker that already is being used to detect alcohol consumption is a compound called ethyl glucuronide (EtG). This compound, which is formed in the liver by linking ethanol with glucuronic acid, remains detectable in serum, plasma, and hair for days after alcohol consumption. Several traditional experimental strategies already are being used to detect EtG for forensic purposes. Recently, however, researchers demonstrated that EtG also can be detected in liver extracts from rats exposed to alcohol for 4 days using a metabolomic approach (i.e., proton NMR) (Nicholas et al. 2006). This study showed that proton NMR is capable of detecting EtG and that future NMR-based metabolomic studies of alcohol consumption could integrate measurements of this metabolite with other NMR-based measurements made simultaneously on the same biological samples to correlate alcohol consumption with other metabolic alterations.

## Concluding Remarks

The most important value that metabolomics may add to alcohol-associated research is the increased number of individual metabolites within different metabolite classes that can be analyzed, thereby allowing researchers to gain a greater understanding of distinct biochemical processes associated with these metabolites. For example, by maximizing the number of lipid metabolites that can be measured, researchers may be able to more accurately assess the effects of a drug or medication on lipid metabolism compared with using more traditional measurements that measure only a small number of entities (e.g., only cholesterol esters or triglycerides). The LIPIDMAPS initiative should serve to drive further progress in this area. Moreover, as the example of carbohydrate metabolism studies and particularly the SIDMAP study of Gleevec demonstrates, such a systems approach to biochemical analysis of the effects of a specific compound may allow quantitative assessment of two differentially affected pathways (e.g., downregulation of the oxidative branch, and concurrent upregulation of the nonoxidative branch, of the pentose phosphate pathway).

Development of strategies that allow quantitative analyses of diverse classes of metabolites and their interaction also may prove rewarding. Clearly, altered metabolism of one class of metabolites can impinge on the metabolism of another class of metabolites (e.g., dysfunction in lipid metabolism likely is associated with changes in other metabolite classes). An increased understanding of such interactions would help researchers to better understand disease progression in different patient groups, such as the numerous disease subsets among alcohol-dependent patients. For example, studies found that perturbations in lipid metabolism can contribute not only to cardiovascular dysfunction and inflammation but also to cognitive decline (Yaffe et al. 2004). Accordingly, alcohol’s effects on metabolism in the brain may, at least in part, be secondary to or correlate with alcohol’s effect on the liver. Because many biochemical pathways are interactive, studies that address their integration and co-regulation may potentially contribute to a more detailed understanding of disease progression and suggest novel target points for development of new medications.

Traditional biochemical approaches already have provided important contributions to understanding many of the neural, hepatic, and other diseases associated with alcohol dependence. However, the development and application of strategies that maximize the number of metabolites that can be measured in a single experiment may further enhance this understanding, help identify metabolites and metabolic pathways that likely contribute to the progression of alcoholism, help develop effective interventions, and may even lead to individualized treatment regimes. These potential benefits of metabolomic research in many disease areas now are being widely recognized, as shown by the metabolomic components that are part of the NIH Roadmap Initiative and the NIH Genes, Environment and Health Initiative.

## Footnotes

1 For a definition of this and other technical terms, see the glossary on p. 84.

2 The issue of confounding influences is, however, not specific to metabolomics studies such as this well-known example but also can occur in any other type of study.

3 Differential expression of PPARs and SREBPs is observed in many diseases associated with lipid dysregulation, such as diabetes and atherosclerosis.
